# Within- and between-host mathematical modeling of *Mycobacterium avium* subspecies *paratuberculosis* (MAP) infections as a tool to study the dynamics of host-pathogen interactions in bovine paratuberculosis

**DOI:** 10.1186/s13567-015-0205-0

**Published:** 2015-06-19

**Authors:** Ad P Koets, Yrjö T Gröhn

**Affiliations:** Department of Bacteriology and TSE, Central Veterinary Institute, part of Wageningen University and Research Centre, Edelhertweg 15, 8219 PH Lelystad, The Netherlands; Department of Farm Animal Health, Faculty of Veterinary Medicine, Utrecht University, Yalelaan 1, 3584 CL Utrecht, The Netherlands; Department of Population Medicine and Diagnostic Sciences, College of Veterinary Medicine, Cornell University, Ithaca, NY 14853 USA

**Fig. 1:**
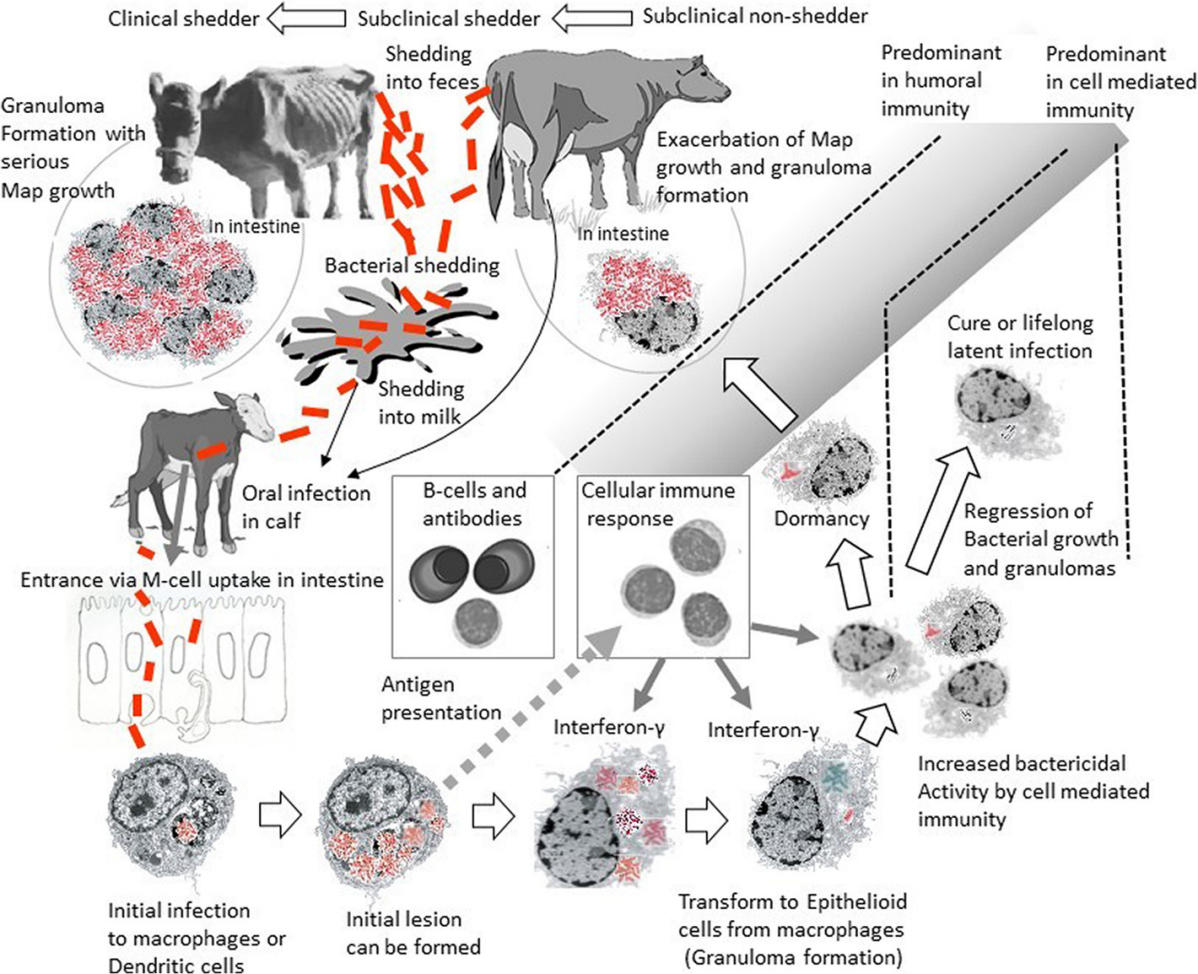
**A schematic representation of the complexities of the within and between host dynamics of** ***Mycobacterium avium*** **subspecies** ***paratuberculosis*** **infection in cattle (courtesy Dr. E. Momotani)**

Paratuberculosis is a chronic intestinal infection of ruminants caused by *Mycobacterium avium* ssp. *paratuberculosis* (MAP). Although a small proportion of animals are able to clear the infection, the majority of exposed animals will become chronically infected for life. Substantial economic losses to the dairy industry are a result of the clinical manifestation of the disease; however, production losses and increased morbidity in subclinically affected animals are also important factors. Only a small fraction (approximately 10%) of chronically infected animals will develop a fatal progressive form of the disease. The progressive form of clinical paratuberculosis is characterized by chronic intractable diarrhea in cattle and weight loss, production losses and severe emaciation leading to death since no treatment is available. A schematic representation of current views regarding the dynamics and pathogenesis of paratuberculosis is depicted in Fig. 1. This special issue of *Veterinary Research* is a compilation of work that explores and connects the dynamics of MAP-host interaction biology at multiple levels. The aim of this work is to highlight the potential of mathematical modelling techniques in exploring knowledge gaps and alternative explanations for observations made in data from experimental and field studies, which may ultimately lead to a better understanding of this complex infection.

In the opening article Koets et al. [1] review the immunobiology of bovine paratuberculosis. The in vivo pathogenesis studies have been largely based on data obtained from cross-sectional studies, mainly with a primary diagnostic focus. These studies have proposed the hypothesis that the pathogenesis of bovine paratuberculosis involves a switch in adaptive immune responses from Type 1 (cellular, IFNγ mediated) to Type 2 (antibody mediated) immune responses potentially indicative of a T helper 1 (Th1) to T helper 2 (Th2) switch in adaptive immune responses. However, the (limited) number of longitudinal studies in cattle as well as sheep have produced inconclusive results with respect to certain immune response patterns (Th1-Th2) explaining differences in bacterial shedding patterns. In addition, this review identified a major knowledge gap with respect to the insight into the spatio-temporal relations between the host immune system and MAP in the different anatomical locations playing a role in the pathogenesis of paratuberculosis. The current ex vivo / in vitro data indicate that monocytes/macrophages are subverted by MAP following infection within a period of 12–24 h into sites of replication due to pronounced down regulation of multiple genes involved in bactericidal responses as well as those crucial in initiating pro-inflammatory (adaptive) immune responses. However, to generate an adaptive (Th1) T cell response, the antigen has to be taken up by professional antigen presenting cells transported to the draining lymph node where interaction with T cells will lead to induction and clonal expansion. Subsequently, effector T cells are released in the circulation and to exert their function they have to home in on the intestinal sections which harbor the infected macrophages. Due to the temporal discrepancy between macrophage subversion and T cell activation the protective efficacy of a Th1 type T cell response dependent on effector CD4 Th1 cells may be restricted and many young granulomatous lesions may escape detection and elimination. Based on estimates of lifespans of tissue macrophages it can be deduced that these granulomas are more dynamic than previously recognized. Within a time span of 3–6 weeks macrophages will probably die and release replicated MAP in the lamina propria which can then start a new cycle of macrophage infection.

Although it has been suggested that MAP-specific immune responses, in particular Th1-type immune responses, are involved in control of MAP replication in cattle, direct evidence in the target species is still lacking. Ganusov et al. [2] analyzed longitudinal data from 20 experimentally infected calves involving frequent measurements of the MAP antigen specific cellular and antibody response and MAP shedding in fecal samples. While the authors found the previously expected positive relationship between the level of shedding and MAP-specific antibody (as detected by ELISA), several additional results are also interesting. In particular, using several different statistical and mathematical modeling techniques, the authors found that in some animals Th1 response is positively associated with the degree of shedding, suggesting that Th1 response may also contribute to pathology in MAP- infected cattle.

As the efficacy of Th1 T cells in the lamina propria may be limited, Klinkenberg et al. [3] explored the extreme variant of this situation in a model paper on the dynamics of granuloma lesions in the absence of an adaptive immune response. They do this by making two mathematical models of the infection, both based on the same simple description of the local infection process, but each with a different assumption on how the infection occupies space within a villus. First, it is assumed that the infection is present in the whole villus and that the local density of macrophages and bacteria changes during the course of the infection, but not the size of the lesion. Second, it is assumed that the macrophages clump together, forming a granuloma, and that the size of the lesion changes during the course of the infection, but not the local density of macrophages inside the lesion. With both models, conditions are identified for growth or containment of lesions, and simulations show that the model can reproduce actually observed shedding patterns in MAP infections. The models thus show that these shedding patterns could be explained by dynamics of the granuloma independent of an adaptive immune response acting on the lesion.

As reviewed by Stevenson [4] in this issue the tools to genotype MAP have exponentially grown in the past 10 years, much supported by the fact that the genome sequence of MAP has become available. These new methods highlight that substantial genomic diversity is present in MAP and this can be used in molecular epidemiology, and to study host adaptation of MAP in different species such as sheep, cattle and wild life. Studies on genomes of MAP have also enabled derivation of its divergence from a recent common ancestor about 10–30,000 years ago. More recently tools have been developed to study functional genomics of MAP under different conditions. These show that MAP adapts to its environment and regulates its gene expression to cope with changing conditions such as intra-cellular survival in macrophages. While data on the influence of host genetic make-up have provided insight into quantitative and qualitative aspects of genetic susceptibility in cattle, limited data are available with regard to the contribution of this genetic variation in the pathogenicity of MAP. Such data as well as the functional genomics of MAP under different conditions are essential for further elucidation of the pathogenesis.

The paper by Schukken et al. [5] describes the data collection systems for a 10-year longitudinal study on MAP in three US dairy farms. The particular value of this data collection system is in the long term follow-up of animals in these farms. Both data and biological materials on infected and non-infected animals were collected and stored in data bases and bio banks. They used these longitudinal data to answer questions on infection biology, infection dynamics and molecular epidemiology of MAP in dairy herds. They argue that longitudinal data provide much more information and value for money compared to repeated cross-sectional data.

Mitchell et al. [6] compared fecal shedding patterns in naturally vs. experimentally infected cattle. The results of this analysis showed that experimental and natural infection have significantly different shedding patterns, indicating that we need to use caution when extrapolating conclusions from experimental studies to field conditions. The analysis of the natural infection data furthermore indicated that intermittent shedders have a low probability of ever becoming high shedders. Finally, the data analysis indicated that infection at a later age leads to less control of infection and significantly higher shedding as compared to being infected as a calf. Many diseases are characterized by a long and varying sub-clinical period. Two main mechanisms can explain such periods: a slow progression toward disease or a sudden transition from a healthy state to a disease state induced by internal or external events.

Louzoun et al. [7] studied epidemiological features of the amount of bacteria shed during bovine MAP infection to test which of these two models, slow progression or sudden transition (or a combination of the two), better explains the transition from intermittent and low shedding to high shedding. Natural infections are not well explained by a slow development of the disease, suggesting a limited within host effect on the sub-clinical to clinical transition. They propose a generic model containing bacterial growth, immune control and the introduction of fluctuations in the bacterial load and shedding through a random noise term in the bacterial dynamics. The noise term represents random fluctuation of for example the growth of a large granuloma, or the effect of external event (weather, diseases, pregnancies, diet) on the bacteria.

This proposed generic model can represent the two hypothesized types of transitions in different parameter regimes. The results show that the sudden sub-clinical to clinical transition model provides a simpler explanation of the data. Environmental fluctuations may be a major element determining the sudden transition to a clinical stage. These conclusions are applicable to a wide variety of diseases, and MAP serves as a good test case based on the large scale measurements of single cow longitudinal profiles in this disease.

Further broadening the scope to the population level, Robins et al. [8] developed an agent-based model for epidemiology of Johne’s disease (JD) in a dairy herd and used the model to analyze cost-effectiveness of a serological test developed by this group. This serological test, a type of enzyme-linked immunosorbent assay (ELISA), has a higher sensitivity than the current commercial ELISA tests. Contact structure and adult infection with the causative agent, MAP, have been missing in many of the previous models for JD epidemiology and are included in this study. Population dynamics analysis showed that prevalence of JD in a dairy farm could be reduced by applying control measures based on the ELISA tests. The effect was more pronounced when the sensitive ELISA was used. Cost-effectiveness of ELISA based control measures was also higher for the sensitive ELISA than for the commercial ELISA.

The paper by Martcheva et al. [9] illustrates a novel way to link a within-host model for MAP with an epidemiological model. The underlying variable in the within-host model is the time since infection. Two compartments, infected macrophages and T cells, of the within-host model feed into the epidemiological model through the direct transmission rate, disease-induced mortality rate, the vertical transmission rate, and the rate of shedding of MAP into the environment. The epidemiological reproduction number depends on the within-host bacteria load in a complex way, exhibiting multiple peaks. Consequently, low within-host bacterial load or shedding does not necessarily imply low epidemiological reproductive number or low prevalence.

Two additional contributions to this special issue provide further evidence regarding dynamics of MAP infection in young calves and young stock. The paper by Eisenberg et al. [10] provides indications that neither exposure of calves on the day of birth to MAP containing colostrum nor the status of the dam at the day of birth are major risk factors for calves to become shedders during the first 2 years of life. Instead, the contaminated environment in which these animals are raised may be a more important driving factor in transmission of infection. The study also provides evidence that shedding of MAP in young stock is not a rare event when raised in a MAP contaminated environment. These notions are further substantiated by the paper by Wolf et al. [11] analysing data from more than 2600 calves and young stock originating from 18 farms with endemic MAP infection. They show that up to 8.1% of young stock had MAP positive faeces. Combined, the final two contributions indicate that the within-host dynamics of MAP infection in calves and young stock is an active process and should not be considered a latent phase despite lack of positivity in immunological diagnostic assays. In addition these data also suggest a more prominent role for calves as sources of environmental contamination and within-herd transmission of MAP on dairy farms with endemic MAP infection.

In conclusion, mycobacterial diseases such as paratuberculosis are extremely difficult to control due to the complexities arising from the incompletely understood pathogenesis, poor (immunological) diagnostic sensitivity, environmental and wildlife reservoirs of infection, heterogeneous strain infectiousness and host susceptibility to infection. The key to controlling these and similar diseases is an integrated approach to understanding the pathways through which pathogen transmission occurs at all levels in an ecosystem: within animals, between individual animals of various ages, between livestock and wildlife, and between livestock and the environment. As we are studying agricultural systems, which must be commercially viable, economic decisions play an important role in contact structures, cattle life histories, and control measures. Thus, we must consider the effects of economic determinants on the transmission dynamics of these systems, as well. Traditionally, a single discipline approach fails to consider the system as a whole. We believe that an ecological approach, simultaneously considering the impact of all aspects of the disease ecosystem, combined with economic analysis, will offer many advantages over past approaches. Mathematical models can be a valuable tool in hypothesis testing and the analysis of such complex systems and aid in understanding the complexities involved in efficacious control of chronic persistent infections such as bovine paratuberculosis. These tools will help us to predict the role of each potential source of infection and to recommend control options targeting these sources, expanding the toolbox available to decision makers.
